# Development of a Computationally Efficient CFD Method for Blood Flow Analysis Following Flow Diverter Stent Deployment and Its Application to Treatment Planning

**DOI:** 10.3390/bioengineering12080881

**Published:** 2025-08-19

**Authors:** Soichiro Fujimura, Haruki Kanebayashi, Kostadin Karagiozov, Tohru Sano, Shunsuke Hataoka, Michiyasu Fuga, Issei Kan, Hiroyuki Takao, Toshihiro Ishibashi, Makoto Yamamoto, Yuichi Murayama

**Affiliations:** 1 Department of Mechanical Engineering, Tokyo University of Science, Tokyo 125-8585, Japan; yamamoto@rs.tus.ac.jp; 2 Division of Innovation for Medical Information Technology, The Jikei University School of Medicine, Tokyo 105-8461, Japan; 4523522@ed.tus.ac.jp (H.K.); takao@jikei.ac.jp (H.T.); 3 Graduate School of Mechanical Engineering, Tokyo University of Science, Tokyo 125-8585, Japan; 4 Department of Neurosurgery, The Jikei University School of Medicine, Tokyo 105-8461, Japan; kostadinkaragiozov@yahoo.com (K.K.); t.sano23@gmail.com (T.S.); s.hataoka@jikei.ac.jp (S.H.); fugamichiyasu@gmail.com (M.F.); isseikan@gmail.com (I.K.); t-ishibashi@jikei.ac.jp (T.I.); ymurayama@jikei.ac.jp (Y.M.)

**Keywords:** intracranial aneurysm, flow diverter stent, hemodynamics, computational fluid dynamics, porous media model

## Abstract

Intracranial aneurysms are a serious cerebrovascular condition with a risk of subarachnoid hemorrhage due to rupture, leading to high mortality and morbidity. Flow Diverter Stents (FDSs) have become an important endovascular treatment option for unruptured large or wide-neck aneurysms. Hemodynamic factors significantly influence treatment outcomes in aneurysms treated with FDSs, and Computational Fluid Dynamics (CFD) has been widely used to evaluate post-deployment flow characteristics. However, conventional wire-resolved CFD methods require extremely fine meshes to reconstruct individual FDS wires, resulting in prohibitively high computational costs. This severely limits their feasibility for use in clinical treatment planning, where fast and robust simulations are essential. To address this limitation, we developed a computationally efficient CFD method that incorporates a porous media model accounting for local variations in wire density after FDS deployment. Based on Virtual Stent Simulation, the FDS region was defined as a hollow cylindrical domain with spatially varying resistance derived from cell-specific wire density. We validated the proposed method using 15 clinical cases, demonstrating close agreement with conventional wire-resolved CFD results. Relative errors in key hemodynamic parameters, including velocity, shear rate, inflow rate, and turnover time, were within 5%, with correlation coefficients exceeding 0.98. The number of grid elements, the data size, and total analysis time were reduced by over 90%. The method also allowed comparison between Total-Filling (OKM Grade A) and Occlusion (Grade D) cases, and evaluation of different FDS sizing, positioning, and coil-assisted strategies. The proposed method enables practical and efficient CFD analysis following FDS treatment and supports hemodynamics-based treatment planning of aneurysms.

## 1. Introduction

Intracranial aneurysms are a cerebrovascular disorder in which part of a cerebral artery bulges into a sac-like shape, affecting approximately 3.2% of the general population [1]. When aneurysm rupture occurs, it results in subarachnoid hemorrhage (SAH). SAH carries a poor prognosis: about 12% of patients die before reaching a medical facility, and in-hospital mortality has been reported at approximately 32% [2,3]. Moreover, nearly half of those who survive are left with permanent neurological or cognitive deficits, making return to their premorbid level of function particularly challenging [2,4]. Consequently, when an unruptured aneurysm is identified, neurosurgical interventions, including endovascular treatment, are considered. In recent years, Flow Diverter Stents (FDSs) have been widely used in endovascular treatment for aneurysms. An FDS typically consists of 48–96 braided metallic wires, each about 20–50 μm in diameter, forming a straw-like structure [5,6,7]. It is deployed into the target intracranial artery using a micro-catheter. Due to its high metallic coverage, an FDS reduces the blood inflow into the aneurysm and promotes neointimal formation along the device surface, ultimately resulting in aneurysm occlusion and reducing the risk of rupture [8,9,10,11,12,13,14]. However, complete aneurysm occlusion is not always achieved, and residual blood flow can persist even after FDS deployment [15,16]. Occlusion status is commonly evaluated using the O’Kelly–Marotta (OKM) Grading Scale, and previous studies have reported hemodynamic differences between Grade A (Total-Filling cases) and OKM Grade D (Occlusion cases) [17,18,19]. This suggests that changes in hemodynamics before and after FDS deployment are critical to treatment outcome.

Computational fluid dynamics (CFD) analysis of these changes may facilitate more effective treatment planning, and has therefore been widely applied to investigate hemodynamic factors in cases treated with FDS deployment [20,21,22,23,24]. CFD analysis requires mesh generation. Yet, while FDS wires measure within the order of tens of micrometers, the involved arteries are usually a few millimeters in diameter. Consequently, conventional CFD approaches that precisely model each individual wire demand a very fine mesh conforming to the wire diameter, often resulting in hundreds of millions of elements [18,25,26]. This leads to prohibitively high computational costs, undermining the feasibility of using CFD as a treatment-planning tool. One proposed solution is to approximate the FDS wire region with a porous media model; however, existing methods may suffer from insufficient accuracy due to simplifications, and often depend on specific solver functions [24,27,28,29]. Furthermore, there has been limited discussion on how to utilize these approaches in actual treatment planning.

The objective of this study is to develop and validate a versatile, generalizable, and computationally efficient CFD method for analyzing post-FDS hemodynamics, by incorporating a porous media model capable of accounting for local variations in wire density, and to evaluate its potential clinical utility for treatment planning. We compared the results of this newly developed method with those of a conventional technique, assessing both its validity and its ability to reduce computational cost. We then applied the proposed method to a clinically oriented scenario to evaluate its potential utility in treatment planning. The novelty of this study lies in the development of a local density porous model (LDPM) that integrates spatial variations in wire density derived from Virtual Stent Simulation. In contrast to conventional porous models with uniform or surface-based resistance, our method represents the stent region as a finite-thickness cylindrical domain with cell-specific resistance coefficients. This enables robust and solver-independent CFD analysis of post-FDS hemodynamics. By balancing computational efficiency and analytical accuracy, the proposed method provides a practical tool for integrating CFD into clinical decision-making for aneurysm treatment.

## 2. Materials and Methods

### 2.1. Case Selection

This study was approved by the Ethical Committee of the Jikei University School of Medicine, No. 32-355 (10442), and was conducted in accordance with the Declaration of Helsinki. Between 2016 and 2021, we retrospectively identified 49 patients at the Jikei University School of Medicine who had unruptured aneurysms located in an internal carotid artery and were treated with a Pipeline Embolization Device (PED; Medtronic, Irvine, CA, USA). Patients were excluded based on the following criteria: (i) deployment of more than one FDS for an aneurysm (7/49), (ii) coil-assisted treatment (21/42), and (iii) insufficient imaging resolution for CFD analysis (6/21). As a result, 15 cases were included in the final analysis. For each eligible case, we collected data on patient characteristics, aneurysm location and morphology, details of the deployed Pipeline Embolization Device (type, size, and length), and the O’Kelly–Marotta (OKM) grading scale at six months post-treatment [17]. From these cases, one neurosurgeon, blinded to all other details, randomly selected a case with OKM Grade A and a case with OKM Grade D, which were designated as the Illustrative Total-Filling Case and Illustrative Occlusion Case, respectively.

### 2.2. Outline of the Analysis and Development

**Figure 1:**
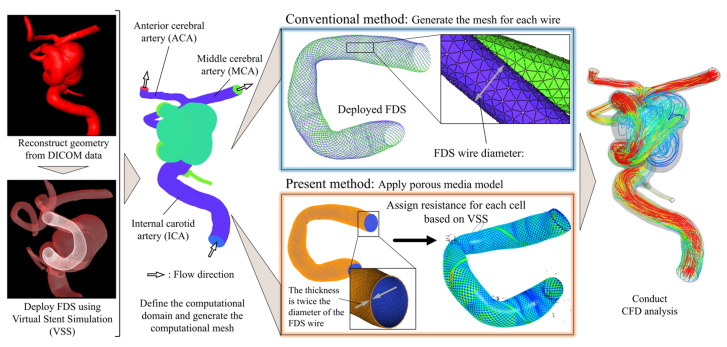
Schematic diagram of the present and conventional CFD analysis methods for aneurysm with an FDS.

Figure 1 provides an overview of the analysis procedures in this study. First, we reconstructed vascular models from DICOM data obtained by three-dimensional digital subtraction angiography (3D-DSA) immediately before FDS deployment. Using these vascular models, we conducted FDS deployment simulations with our in-house program, which incorporates a previously reported Virtual Stent Simulation technique [30]. This simulation computes the local FDS wire configuration by combining information on vessel centerlines and cross-sectional diameters at fixed intervals with the stent’s design specifications, thereby reproducing the geometric distribution of wires once deployed in the artery. We initially performed a conventional CFD analysis. Specifically, we generated a three-dimensional model of the FDS by reconstructing each wire from the Virtual Stent Simulation. Because the wire diameter was 30 μm, we generated a mesh with a maximum element size of 8 μm near the wires, based on previous studies, and conducted CFD analysis under the conditions described later [25]. We also performed CFD analysis using the present method. From the FDS deployment simulation, we defined the wire region as a hollow cylindrical region with a thickness equal to twice the wire diameter (60 μm). This thickness was chosen to reflect the braided structure of the FDS, in which multiple wires intersect and overlap, forming regions with an effective thickness approximately equal to twice the wire diameter. Thus, all wires are geometrically confined within this cylindrical domain, making it a reasonable representation of the physical extent of the device. A computational grid was created for this cylindrical region, where the maximum grid size was varied at 0.05, 0.1, 0.2, and 0.4, to examine grid dependence. A porous media model with spatially varying resistance (LDPM) was then assigned to capture local variations in wire density, as described below. The regions outside the FDS wire area were discretized according to previous studies, and CFD analysis was conducted under the same conditions as in the conventional approach [31]. ANSYS ICEM CFD 2020 R1 (ANSYS Inc., Canonsburg, PA, USA) was used for all mesh generation. The size and position of the deployed PED were determined according to the actual clinical procedure. For the Illustrative Total-Filling Case, we performed additional analyses using alternative PED sizes and deployment positions, reflecting the preoperative considerations discussed prior to the treatment. We also examined scenarios assuming coil-assisted treatment, which had not been performed clinically. In these scenarios, we considered deployment of one or two coils using Target XL 360 Soft 7 × 20 (Stryker Neurovascular, Fremont, CA, USA). To account for the flow resistance introduced by the coils, we applied a porous media model within the aneurysm region, following methods reported in previous studies [32,33].

### 2.3. Development of LDPM for FDSs

For the porous media model applied to the hollow cylindrical region representing the FDS wires, we employed a screen-based porous model, as described by Idelchik et al. [34], which is based on the approach proposed by Abdehkakha et al. [29]. In this model, the pressure loss Δ*p* across a wire mesh is expressed as

(1) $$∆p=-\frac{\rho }{2}\left[1.3\left(1-\varepsilon \right)+{\left(\frac{1}{\varepsilon }-1\right)}^{2}\right]v^{2}-\frac{11\mu }{D_{h}}v$$

where *ρ* is the fluid density, *v* is the velocity, *μ* is the fluid viscosity, *ε* is the porosity, and *D_h_* is the hydraulic diameter. Moreover, *ε* and *D_h_* are defined as follows:

(2) $$\varepsilon =\frac{A_{p}}{A_{t}}=\frac{A_{t}-A_{w}}{A_{t}}$$

(3) $$D_{h}=A_{t}\sin\alpha$$

where *A_p_* is the pore area, *A_t_* is the total area, *A_w_* is the wire area, and *α* is the braiding angle [29]. These quantities are associated with each cell (one rhombus unit) formed by the FDS wires. In this study, we calculated Equations (2) and (3) for each cell based on the Virtual Stent Simulation results, and applied the resulting pressure loss from Equation (1) to the centroid of each cell. Furthermore, we set the resistance coefficient of the wire intersection regions on the cylindrical surface to zero. Hence, our model can reflect local variations in wire density within the FDS as a porous media model.

### 2.4. Analysis Conditions

Blood flow analyses were conducted using ANSYS CFX 2020 R1 (ANSYS Inc., Canonsburg, PA, USA), following a previously developed and validated method [31]. Specifically, the flow field was assumed as a three-dimensional, incompressible laminar flow, and blood was modeled as a non-Newtonian fluid with a density of 1100 kg/m^3^ [35]. This non-Newtonian model was based on viscosity measurements obtained via a falling needle viscometer on whole-blood samples from 12 patients with aneurysms [36,37]. To minimize the influence of inlet and outlet boundary conditions, 75 mm extension tubes were added to all inlets and outlets, with boundary conditions applied at the ends of these tubes. Steady flow without pulsation was assumed, and a mass flow rate of 0.003465 kg/s, corresponding to diastolic conditions of the internal carotid artery in healthy adults, was imposed at the inlet. A static pressure of 0 Pa was specified at the outlet, and no-slip boundary conditions were applied to the arterial wall and stent-wire surfaces, which were modeled as rigid. From the CFD results, we calculated the mean velocity within the aneurysm, the mean shear rate at the aneurysm wall, the inflow rate into the aneurysm, and the theoretical turnover time of blood within the aneurysm. The theoretical turnover time was determined by dividing the aneurysm volume by the inflow rate. For each of these parameters, we then calculate the relative error, using the following equation:

(4) $$X_{RE}\;=\;\frac{X_{LDPM}-X_{VS}}{X_{VS}}\times 100$$

where *X_L__DPM_* is the value obtained using the present method and *X_VS_* is the value derived from the conventional method.

## 3. Results

### 3.1. Clinical Overview of the Selected Cases

A total of 49 aneurysms were enrolled. Of these, cases involving more than one FDS deployment for an aneurysm (7/49), cases treated with coil-assisted treatment (21/42), and cases without images of sufficient resolution for CFD analysis (6/21) were excluded, leaving 15 aneurysms in 15 patients for analysis. All 15 aneurysms were located in the internal carotid artery, and 11 patients (73.3%) were female. The mean patient age was 63.1 ± 10.6 years. The mean maximum aneurysm size and neck diameter were 17.4 ± 4.7 mm and 8.5 ± 2.0 mm, respectively. At six months follow-up, the O’Kelly–Marotta (OKM) Grading Scale distribution was as follows: Grade A in 2 cases, Grade B in 3, Grade C in 5, and Grade D in 5. The Illustrative Total-Filling Case was an aneurysm measuring 14.5 mm in width, 14.8 mm in height, and 4.8 mm in neck diameter, treated with a Pipeline 4.5 × 20 device.

### 3.2. Grid Independence in CFD Analysis

**Figure 2:**
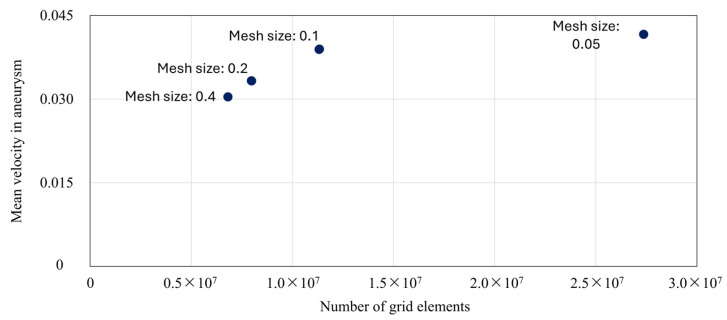
Grid dependency of the present method for mean velocity in the aneurysm.

To investigate the grid dependency for the velocity in the aneurysm for the present method, we performed CFD analysis for the Illustrative Occlusion Case under varying grid sizes in the porous media region. Figure 2 plots the total number of mesh elements on the horizontal axis against the mean velocity in the aneurysm on the vertical axis. As shown, the results converge when the mesh size is 0.1 or smaller. Based on these findings, and taking both computational cost and accuracy into account, we adopted a mesh size of 0.1.

### 3.3. Comparison with Conventional Method

**Figure 3:**
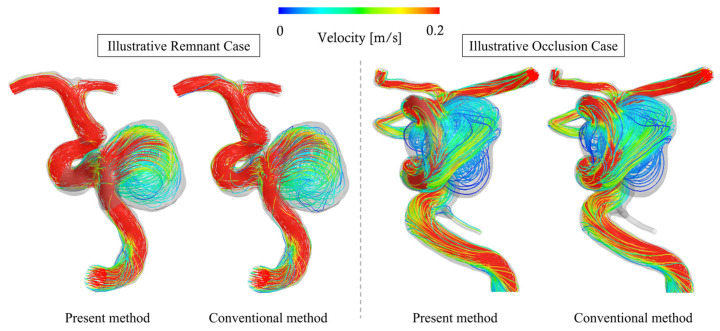
Comparison of streamlines between the present and conventional methods in the Illustrative Total-Filling and Occlusion Case.

**Figure 4:**
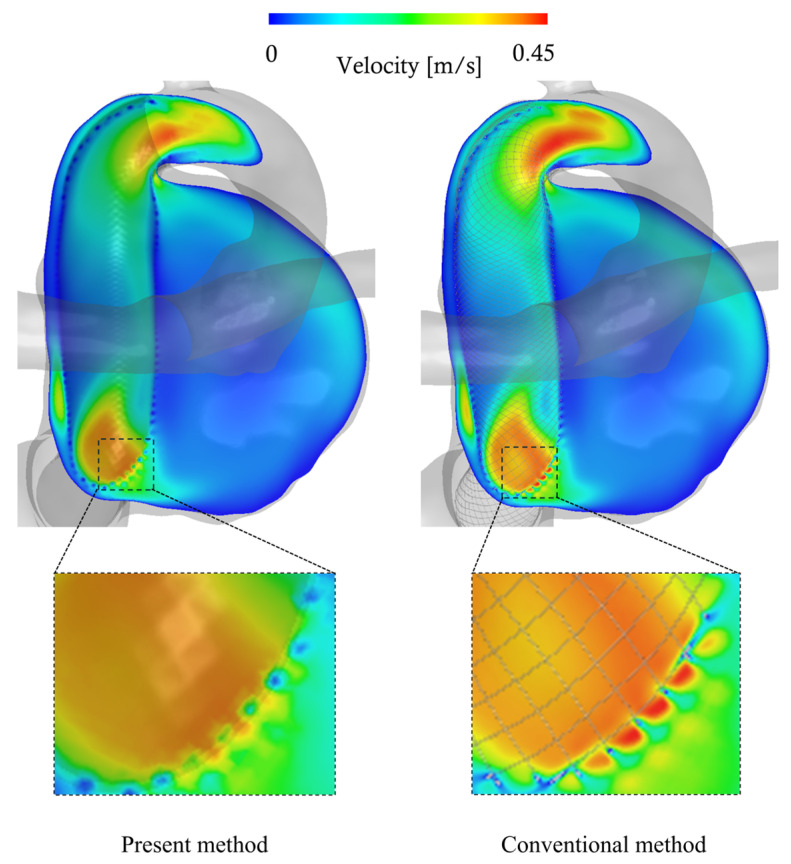
Flow field comparison between the present and conventional methods on the cross-sectional plane of the aneurysm and parent artery, and around the stent wires.

**Figure 5:**
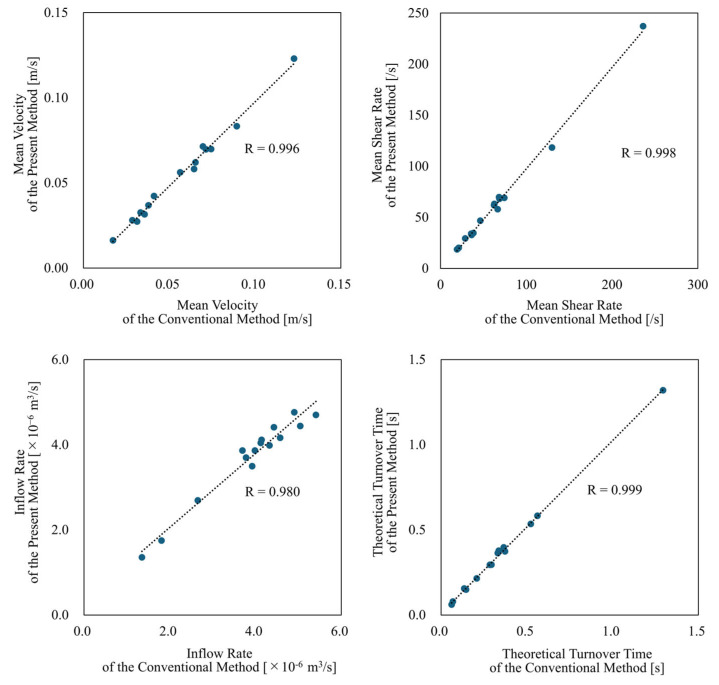
Scatter plots and correlation coefficients of hemodynamic parameters derived from the present method and the conventional method.

For the Illustrative Total-Filling Case and Illustrative Occlusion Case, Figure 3 shows the streamlines computed by both the conventional method and the present method. Additionally, Figure 4 presents cross-sectional velocity distributions of the aneurysm and parent artery in the case that showed the largest discrepancy in inflow (13.1%) between the methods among all analyzed cases; flow fields around the stent wires are also illustrated. From these visualizations, the overall flow fields produced by both methods appear qualitatively similar. However, near the stent wires, the results differ. Specifically, in the conventional method, blood flow passing through the FDS mesh narrows due to interaction with the wires, leading to a temporary increase in velocity, whereas this phenomenon was not observed in the present method. This discrepancy is expected, because porous media models represent bulk flow resistance and do not capture individual flow channels between wires. As a result, local acceleration effects such as jet-like velocity peaks caused by flow constriction in the wire mesh are inherently smoothed out in the porous domain. Although this limits the resolution of near-wire flow patterns, it does not significantly affect the overall hemodynamic parameters evaluated in this study. For quantitative evaluation, we generated scatter plots of each parameter as derived from each method, along with their correlation coefficients (see Figure 5). All parameters showed a significantly positive correlation (R > 0.98). Table 1 summarizes the mean values of each parameter for both methods, as well as their mean relative errors. The mean velocity, mean shear rate, and inflow rate tended to be underestimated by approximately 4%, whereas the theoretical turnover time was overestimated by about 5%. Table 1 also represents the mean number of grid elements and data file sizes for both methods, together with the rate of change. Adopting the present method reduced the mean number of mesh elements from about 200 million to approximately 7 million, a decrease of over 96%. Similarly, the data file sizes of the results were reduced by more than 95%, on average. Moreover, while the conventional method generally took around 57 h to process one case, the present method completed the analysis in about 4 h, indicating a reduction of approximately 93%.

### 3.4. Comparison Between Total-Filling (OKM Grade A) and Occlusion (OKM Grade D) Cases

**Figure 6:**
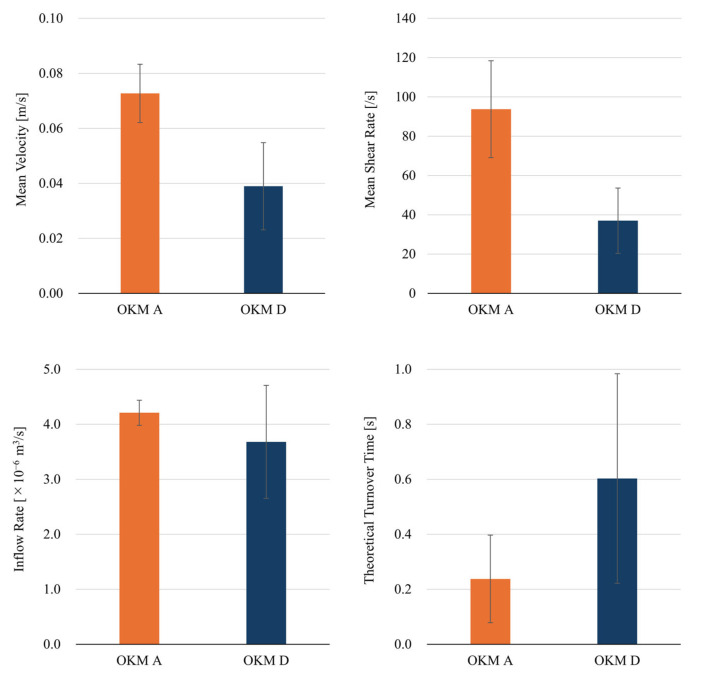
Comparison of hemodynamic parameters between OKM Grade A (Total-Filling) and OKM Grade D (Occlusion).

Using the present method, we calculated the mean values of the hemodynamic parameters for the two OKM Grade A cases and the five OKM Grade D cases (see Figure 6). Comparisons reveal that the mean velocity, mean shear rate, and inflow rate in OKM Grade A cases were generally higher than those in OKM Grade D cases, while the theoretical turnover time was longer in OKM Grade D cases. In other words, Occlusion cases (OKM Grade D) exhibited lower velocity, shear rate and inflow, and longer theoretical turnover time, compared with Total-Filling cases (OKM Grade A). Notably, the mean shear rate and theoretical turnover time differed by more than a factor of 2.5. In the Illustrative Total-Filling Case, the mean velocity, mean shear rate, inflow rate, and theoretical turnover time were 0.06 m/s, 69.1 s^−1^, 4.0 × 10^−6^ m^3^/s, and 0.40 s, respectively. In contrast, those in the Illustrative Occlusion Case were 0.04 m/s, 29.4 s^−1^, 3.7 × 10^−6^ m^3^/s, and 0.53 s, respectively.

### 3.5. Evaluation of the Impact of FDS Size and Deployment Position

For the Illustrative Total-Filling Case, we performed CFD analyses with the present method, under various conditions involving alternative PED sizes and deployment positions that were originally considered during preoperative planning (see Table 2). Two potential deployment positions (Position A and Position B) were discussed for the Pipeline 5.0 × 20 device, and the analyses were conducted for each of them. The results for each parameter were compared with those under the original condition, which reflected the data related to the stent size and position actually applied in clinical settings. Compared to the original configuration, Position A produced changes of 3.0%, 3.0%, 1.7%, and −1.7% for mean velocity, mean shear rate, inflow rate, and theoretical turnover time, respectively. In contrast, the corresponding rates of change for Position B were 4.8%, 5.5%, 6.0%, and −5.7%, respectively.

### 3.6. Effects of Coil-Assisted Treatment on the Illustrative Total-Filling Case

Table 2 shows the CFD analysis results assuming FDS coil-assisted treatment in the Illustrative Total-Filling Case. The simulated coil used was the Target XL 360 Soft 7 × 20 (Stryker Neurovascular, Fremont, CA, USA). The volume embolization ratio (VER), calculated based on the aneurysm volume, was 1.2% with a single coil and 2.4% with two coils. Compared to the Original condition, the relative changes with one coil were −45.4% in mean velocity, −46.0% in mean shear rate, −0.3% in inflow rate, and 0.3% in theoretical turnover time. With two coils, the corresponding changes were −63.1%, −63.0%, 0.7%, and −0.7%, respectively.

## 4. Discussion

In this study, we demonstrated that the present new method enables CFD analysis of aneurysms treated with FDS at a substantially reduced computational cost compared to the conventional method, while maintaining generally consistent analytical results. Previous studies have reported that aneurysm occlusion after FDS deployment is influenced by hemodynamic factors such as mean velocity, shear rate, and blood turnover time in aneurysm [19]. Consistent with these findings, our comparison between OKM Grade A (Total-Filling) and OKM Grade D (Occlusion) cases also suggested a potential relationship between hemodynamics and aneurysm occlusion. Despite the methodological differences, discrepancies in the hemodynamic parameters between the two approaches remained within approximately 5%, and the overall flow fields were qualitatively similar. However, differences were observed regarding how each method represented blood flow through the stent mesh. In the conventional method, flow through the FDS mesh was transiently accelerated due to the narrowed flow paths and elevated pressure from wire-induced collisions. This phenomenon was not fully replicated by the present method. Nevertheless, even in the case with the largest discrepancy in inflow rate, the overall flow patterns in the aneurysm and parent artery were qualitatively similar. This indicates that these local hemodynamic differences immediately downstream of the mesh may have limited influence on the broader flow field.

Importantly, our method achieved reductions exceeding 90% in total analysis time, mesh element count, and CFD result file size. To address the computational burden associated with CFD analysis of FDS-treated aneurysms, previous studies have proposed using uniform porous media models with a constant resistance coefficient applied to the FDS wire region [24,27]. However, such approaches overlook the anisotropic mesh density resulting from vessel curvature and diameter variation, which alters local wire density (e.g., higher on the inner curve, lower on the outer curve). Consequently, models assuming uniform resistance throughout the stent are insufficient for accurate hemodynamic simulation. Abdehkakha et al. proposed an inhomogeneous porous medium (iPM) model that incorporates local wire density information obtained from FDS deployment simulations [29]. While effective, their method applies a porous media model to a zero-thickness surface and depends on solver-specific functionalities, which limits its broader applicability. Recent studies have also attempted to enhance porous models for hemodynamic evaluation in aneurysms, but the challenges regarding spatial resolution and solver-dependence remain unresolved [38]. In contrast, our method treats the FDS wire region as a hollow cylindrical domain with finite thickness, making it solver-independent and thus more versatile.

Moreover, by substantially reducing the computational cost required to analyze post-FDS hemodynamics, our method makes it more feasible and closer to daily clinical practice to estimate treatment outcomes or to assess the hemodynamic changes arising from FDS or coil deployment, compared with conventional methods. In this study, we explored this utility by comparing OKM Grade D (Occlusion) and Grade A (Total-Filling) cases at six-month follow-up. The findings suggested that reduced inflow and prolonged turnover time may contribute to aneurysm occlusion, supporting prior evidence with a new easier-to-perform methodology [19]. Although our analysis included only two Grade A cases and five Grade D, the reduced computational burden of the present method may allow future analyses involving larger patient cohorts, potentially facilitating further identification of hemodynamic predictors of treatment outcomes. Furthermore, we employed the present method to simulate multiple treatment scenarios, including changes in FDS size and position, as well as the addition of coil-assisted treatment, and quantitatively assessed hemodynamic changes relative to the original condition. These simulations quantitatively captured the hemodynamic differences in each scenario, providing valuable decision-making indices for FDS-based treatments. For example, in the Illustrative Total-Filling Case, changing stent size or position resulted in a maximum of 6% change in velocity, shear rate, inflow, or turnover time, indicating minimal influence on flow suppression. By contrast, coil deployment substantially reduced the velocity and shear rate, with additional reduction upon deploying more coils. Inflow and turnover time remained largely unchanged, as these parameters were measured at the aneurysm neck, upstream of the coils. These findings suggest that, in this particular case, coil-assisted treatment might have been a more appropriate choice than modifying FDS parameters alone. Not so many prior studies have demonstrated how the simulation approach could be directly applied to treatment planning; hence, these findings provide renewed support for the clinical application of our method.

Nevertheless, this study has some limitations. First, all analyses were performed under steady-flow conditions, and the validity of our method under pulsatile (unsteady) flow has not been thoroughly verified. In the conventional approach, large grid sizes result in extremely high computational cost per time step, making unsteady simulations impractical. However, because the present method substantially reduces the number of grid elements, it is theoretically more suitable for pulsatile-flow analysis. Future work should thus include validating accuracy under unsteady-flow conditions. In addition, as in many previous studies, we did not use patient-specific blood properties, such as individualized density or viscosity, nor did we apply personalized inlet and outlet boundary conditions. Moreover, although we conducted case-based analyses with a view toward potential application as a treatment planning tool, the comparison between Occlusion and Total-Filling cases was limited to five cases with OKM Grade D and two cases with OKM Grade A. In addition, modifications to FDS size and position, as well as the use of coil-assisted treatment, were examined in only a single Illustrative Total-Filling Case. Applying the proposed method to a larger number of cases is expected to clarify the hemodynamic influences on individual clinical courses following FDS deployment. In particular, by comparing cases that resulted in OKM Grade A (Total-Filling) and Grade D (Occlusion) during follow-up, it may become possible to identify key hemodynamic features that determine treatment outcomes and to predict patient-specific outcomes prior to treatment. Furthermore, in cases where complete occlusion is unlikely to be achieved with FDS alone, this method could be used to simulate coil-assisted treatment scenarios and to design specific treatment strategies, including the optimal number of coils. In terms of clinical implementation, the proposed method may be integrated into neurosurgical workflows as a preoperative planning tool. Because it uses vascular models reconstructed from routinely acquired 3D-DSA images and does not require detailed manual modeling of stent geometry, it can be incorporated with minimal disruption to existing imaging and planning pipelines. For example, it could be used during treatment conferences to compare different FDS sizes, deployment positions, or coil-assisted strategies based on simulated hemodynamic outcomes. With further automation and clinical validation, this approach could be incorporated into clinical platforms to support individualized, hemodynamics-informed decision-making in the treatment of intracranial aneurysms.

## 5. Conclusions

In this study, we developed and validated a CFD-based method for analyzing post-FDS intracranial aneurysms that reduces computational time and cost compared with conventional methods, while maintaining a comparable level of accuracy. By applying this approach, simulating the hemodynamics of post-FDS or coil-assisted treatments becomes more efficient and applicable in clinical settings. Moreover, the method generates quantitative indicators such as flow velocity, shear rate, and turnover time in an aneurysm, which can be incorporated into treatment plans that involve comparatively different types or quantities of devices. Future work will include conducting unsteady-flow analyses and testing the method on a wider range of cases, which will provide further validation and enable more detailed treatment planning strategies.

## Figures and Tables

**Table 1:** Comparison of Hemodynamic Parameters, Number of Grid Elements, and Data Size between Conventional Method and Present Method.

		Conventional Method	Present Method	Relative Error or Reduction [%]
Mean Velocity	[m/s]	0.056 ± 0.027	0.054 ± 0.027	−4.03 ± 4.56
Mean Shear Rate	[/s]	66.3 ± 52.7	64.1 ± 52.5	−3.77 ± 4.78
Inflow Rate	[×10^−6^ m^3^/s]	3.88 ± 1.10	3.69 ± 0.973	−4.13 ± 5.17
Theoretical Turnover Time	[s]	0.352 ± 0.288	0.366 ± 0.293	−5.04 ± 5.19
Number of Grid Elements	[×10^−6^]	213.2 ± 65.9	7.44 ± 2.24	−96.4 ± 1.07
Data Size	[GB]	11.1 ± 3.32	0.526 ± 0.162	−95.1 ± 1.55

**Table 2:** Hemodynamic Changes Resulting from Different Treatment Strategies in the Illustrative Total-Filling Case.

		Original with Pipeline 4.5 × 20	Position A with Pipeline 5.0 × 20 (Change Rate)		Position B with Pipeline 5.0 × 20 (Change Rate)		Pipeline 4.5 × 20 with one Coil (Change Rate)		Pipeline 4.5 × 20 with two Coils (Change Rate)	
Mean Velocity	[m/s]	0.070	0.072	(3.0 [%])	0.073	(4.8 [%])	0.038	(−45.4 [%])	0.026	(−63.1 [%])
Mean Shear Rate	[/s]	77.6	79.9	(3.0 [%])	81.9	(5.5 [%])	41.9	(−46.0 [%])	28.7	(−63.0 [%])
Inflow Rate	[×10^−6^ m^3^/s]	3.29	3.35	(1.7 [%])	3.49	(6.0 [%])	3.28	(−0.3 [%])	3.31	(0.7 [%])
Theoretical Turnover Time	[s]	0.520	0.511	(−1.7 [%])	0.490	(−5.7 [%])	0.521	(0.26 [%])	0.516	(−0.7 [%])

## Data Availability

All data supporting the findings of this study are included in the article. Additional data that are not directly presented in the manuscript are available from the corresponding author upon reasonable request. Some restrictions may apply, due to privacy concerns related to patient data.

## Abbreviations

The following abbreviations are used in this manuscript:

CFD	Computational Fluid Dynamics
DSA	Digital Subtraction Angiography
FDS	Flow Diverter Stent
iPM	inhomogeneous porous medium
LDPM	Local Density Porous Model
OKM	O’Kelly–Marotta
PED	Pipeline Embolization Device
SAH	subarachnoid hemorrhage
VER	Volume Embolization Ratio

## References

[1] Vlak M.H., Algra A., Brandenburg R., Rinkel G.J. (2011). Prevalence of unruptured intracranial aneurysms, with emphasis on sex, age, comorbidity, country, and time period: A systematic review and meta-analysis. Lancet Neurol..

[2] Connolly E.S., Rabinstein A.A., Carhuapoma J.R., Derdeyn C.P., Dion J., Higashida R.T., Hoh B.L., Kirkness C.J., Naidech A.M., Ogilvy C.S. (2012). Guidelines for the management of aneurysmal subarachnoid hemorrhage: A guideline for healthcare professionals from the American Heart Association/American Stroke Association. Stroke.

[3] Schievink W.I., Wijdicks E.F., Parisi J.E., Piepgras D.G., Whisnant J.P. (1995). Sudden death from aneurysmal subarachnoid hemorrhage. Neurology.

[4] Al-Khindi T., Macdonald R.L., Schweizer T.A. (2010). Cognitive and functional outcome after aneurysmal subarachnoid hemorrhage. Stroke.

[5] Ma Y., Krepuska M., Madjidyar J., Schubert T., Thurner P., Kulcsar Z. (2024). Ongoing geometric remodeling of the parent artery after flow-diverter stent reconstruction in cerebral aneurysms: The device design matters. World Neurosurg..

[6] Vivanco-Suarez J., Mendez-Ruiz A., Farooqui M., Bekelis K., Singer J.A., Javed K., Altschul D.J., Fifi J.T., Matsoukas S., Cooper J. (2023). Safety and efficacy of the Surpass Streamline for intracranial aneurysms (SESSIA): A multi-center US experience pooled analysis. Interv. Neuroradiol..

[7] Paliwal N., Yu H., Xu J., Xiang J., Siddiqui A., Yang X., Li H., Meng H. (2016). Virtual stenting workflow with vessel-specific initialization and adaptive expansion for neurovascular stents and flow diverters. Comput. Methods Biomech. Biomed. Engin..

[8] Pierot L., Wakhloo A.K. (2013). Endovascular treatment of intracranial aneurysms: Current status. Stroke.

[9] Starke R.M., Turk A., Ding D., Crowley R.W., Liu K.C., Chalouhi N., Hasan D.M., Dumont A.S., Jabbour P., Durst C.R. (2016). Technology developments in endovascular treatment of intracranial aneurysms. J. Neurointerv. Surg..

[10] Nelson P.K., Lylyk P., Szikora I., Wetzel S.G., Wanke I., Fiorella D. (2011). The Pipeline Embolization Device for the Intracranial Treatment of Aneurysms trial. AJNR Am. J. Neuroradiol..

[11] Kadirvel R., Ding Y.H., Dai D., Rezek I., Lewis D.A., Kallmes D.F. (2014). Cellular mechanisms of aneurysm occlusion after treatment with a flow diverter. Radiology.

[12] Fischer S., Vajda Z., Aguilar Perez M., Schmid E., Hopf N., Bäzner H., Henkes H. (2012). Pipeline embolization device (PED) for neurovascular reconstruction: Initial experience in the treatment of 101 intracranial aneurysms and dissections. Neuroradiology.

[13] Brinjikji W., Murad M.H., Lanzino G., Cloft H.J., Kallmes D.F. (2013). Endovascular treatment of intracranial aneurysms with flow diverters: A meta-analysis. Stroke.

[14] Becske T., Kallmes D.F., Saatci I., McDougall C.G., Szikora I., Lanzino G., Moran C.J., Woo H.H., Lopes D.K., Berez A.L. (2013). Pipeline for uncoilable or failed aneurysms: Results from a multicenter clinical trial. Radiology.

[15] Touzé R., Gravellier B., Rolla-Bigliani C., Touitou V., Shotar E., Lenck S., Boch A.L., Degos V., Sourour N.A., Clarençon F. (2020). Occlusion rate and visual complications with flow-diverter stent placed across the ophthalmic artery’s origin for carotid-ophthalmic aneurysms: A meta-analysis. Neurosurgery.

[16] Hanel R.A., Cortez G.M., Lopes D.K., Nelson P.K., Siddiqui A.H., Jabbour P., Mendes Pereira V., István I.S., Zaidat O.O., Bettegowda C. (2023). Prospective study on embolization of intracranial aneurysms with the Pipeline device (PREMIER study): 3-year results with the application of a flow diverter specific occlusion classification. J. Neurointerv. Surg..

[17] O’Kelly C.J., Krings T., Fiorella D., Marotta T.R. (2010). A novel grading scale for the angiographic assessment of intracranial aneurysms treated using flow diverting stents. Interv. Neuroradiol..

[18] Fujimura S., Brehm A., Takao H., Uchiyama Y., Karagiozov K., Fukudome K., Yamamoto M., Murayama Y., Psychogios M.N. (2022). Hemodynamic characteristics and clinical outcome for intracranial aneurysms treated with the Derivo Embolization Device, a novel second-generation flow diverter. World Neurosurg..

[19] Zhang M., Tupin S., Anzai H., Kohata Y., Shojima M., Suzuki K., Okamoto Y., Tanaka K., Yagi T., Fujimura S. (2021). Implementation of computer simulation to assess flow diversion treatment outcomes: Systematic review and meta-analysis. J. Neurointerv. Surg..

[20] Chong W., Zhang Y., Qian Y., Lai L., Parker G., Mitchell K. (2014). Computational hemodynamics analysis of intracranial aneurysms treated with flow diverters: Correlation with clinical outcomes. AJNR Am. J. Neuroradiol..

[21] Mut F., Raschi M., Scrivano E., Bleise C., Chudyk J., Ceratto R., Lylyk P., Cebral J.R. (2015). Association between hemodynamic conditions and occlusion times after flow diversion in cerebral aneurysms. J. Neurointerv. Surg..

[22] Zhang M., Li Y., Zhao X., Verrelli D.I., Chong W., Ohta M., Qian Y. (2017). Haemodynamic effects of stent diameter and compaction ratio on flow-diversion treatment of intracranial aneurysms: A numerical study of a successful and an unsuccessful case. J. Biomech..

[23] Paliwal N., Jaiswal P., Tutino V.M., Shallwani H., Davies J.M., Siddiqui A.H., Rai R., Meng H. (2018). Outcome prediction of intracranial aneurysm treatment by flow diverters using machine learning. Neurosurg. Focus..

[24] Beppu M., Tsuji M., Ishida F., Shirakawa M., Suzuki H., Yoshimura S. (2020). Computational fluid dynamics using a porous media setting predicts outcome after flow-diverter treatment. AJNR Am. J. Neuroradiol..

[25] Uchiyama Y., Fujimura S., Takao H., Suzuki T., Hayakawa M., Ishibashi T., Karagiozov K., Fukudome K., Murayama Y., Yamamoto M. (2021). Hemodynamic investigation of the effectiveness of a two overlapping flow diverter configuration for cerebral aneurysm treatment. Bioengineering.

[26] Reymond P., Bernava G., Brina O., Hofmeister J., Rosi A., Lövblad K.O., Machi P. (2025). A novel method for brain aneurysms computed fluid dynamics analysis after flow diverter stent implantation based on micro-computed tomography reconstruction. J. Biomech..

[27] Augsburger L., Reymond P., Rufenacht D.A., Stergiopulos N. (2011). Intracranial stents being modeled as a porous medium: Flow simulation in stented cerebral aneurysms. Ann. Biomed. Eng..

[28] Yadollahi-Farsani H., Scougal E., Herrmann M., Wei W., Frakes D., Chong B. (2019). Numerical study of hemodynamics in brain aneurysms treated with flow diverter stents using porous medium theory. Comput. Methods Biomech. Biomed. Engin..

[29] Abdehkakha A., Hammond A.L., Patel T.R., Siddiqui A.H., Dargush G.F., Meng H. (2021). Cerebral aneurysm flow diverter modeled as a thin inhomogeneous porous medium in hemodynamic simulations. Comput. Biol. Med..

[30] Fujimura S., Kan I., Takao H., Uchiyama Y., Ishibashi T., Otani K., Fukudome K., Murayama Y., Yamamoto M. (2021). Development of a virtual stent deployment application to estimate patient-specific braided stent sizes. Annu. Int. Conf. IEEE Eng. Med. Biol. Soc..

[31] Takao H., Murayama Y., Otsuka S., Qian Y., Mohamed A., Masuda S., Yamamoto M., Abe T. (2012). Hemodynamic differences between unruptured and ruptured intracranial aneurysms during observation. Stroke.

[32] Umeda Y., Ishida F., Tsuji M., Furukawa K., Shiba M., Yasuda R., Toma N., Sakaida H., Suzuki H. (2017). Computational fluid dynamics (CFD) using porous media modeling predicts recurrence after coiling of cerebral aneurysms. PLoS ONE.

[33] Fujimura S., Takao H., Suzuki T., Uchiyama Y., Tanaka K., Otani K., Ishibashi T., Fukudome K., Mamori H., Yamamoto M. (2018). Blood flow analysis in coil embolized aneurysms: Difference between porous media and real coil geometry model. Annu. Int. Conf. IEEE Eng. Med. Biol. Soc..

[34] Idelchik I.E. (1986). Handbook of Hydraulic Resistance.

[35] Uchiyama Y., Fujimura S., Takao H., Suzuki T., Ishibashi T., Otani K., Karagiozov K., Fukudome K., Yamamoto H., Yamamoto M. (2022). Role of patient-specific blood properties in computational fluid dynamics simulation of flow diverter deployed cerebral aneurysms. Technol. Health Care.

[36] Yamamoto H., Kawamura K., Omura K., Tokudome S. (2010). Development of a compact-sized falling needle rheometer for measurement of flow properties of fresh human blood. Int. J. Thermophys..

[37] Yamamoto H., Yabuta T., Negi Y., Horikawa D., Kawamura K., Tamura E., Tanaka K., Ishida F. (2020). Measurement of human blood viscosity using Falling Needle Rheometer and the correlation to the Modified Herschel-Bulkley model equation. Heliyon.

[38] Xu J., Karmonik C., Yu Y., Lv N., Shi Z., Liu J.M., Huang Q. (2022). Modeling flow diverters using a porous medium approach: A fast alternative to virtual flow diverter deployment. World Neurosurg..

